# Investigating Morphology and Breakage Evolution Characteristics of Railroad Ballasts over Distinct Supports Subjected to Impact Loading

**DOI:** 10.3390/ma15186295

**Published:** 2022-09-10

**Authors:** Yuanjie Xiao, Yu Jiang, Pan Tan, Kunfeng Kong, Joseph Ali, Ralina Mustafina, Hongwei Zhu, Degou Cai

**Affiliations:** 1 School of Civil Engineering, Central South University, Changsha 410075, China; 2 Ministry of Education (MOE) Key Laboratory of Engineering Structures of Heavy Haul Railway (Central South University), Changsha 410075, China; 3 State Key Laboratory for High-Speed Railway Track Technology, China Academy of Railway Sciences Corporation Limited, Beijing 100081, China

**Keywords:** railway ballast, ballast degradation, impact load, particle breakage, morphology

## Abstract

The ballast bed constantly degrades under the repeated applications of impact loading exerted by passing trains in terms of the particle size, shape, breakage, fouling, etc., thus significantly jeopardizing the in-service performance and operational safety of ballasted tracks. In this study, the morphology and breakage evolution characteristics of railroad ballasts of single- and multiple-size ranges were investigated from laboratory impact-load tests. Both a concrete block and sand layer were placed to mimic the distinct under-ballast supports. The degradation trends of the typical shape and breakage indices were comparatively quantified for different combinations of ballast particle sizes and shapes, under-ballast supports, impact energies, and number of impact-load applications (N). The results show that both shape and size affect ballast particle breakage, with shape being more influential. The breakage severity of flake-like particles is about 1.5–1.66 times and 1.25–1.5 times higher than those of regular and needle-like particles, respectively. Under impact loading, large and small single-size ballasts degrade mainly by breakage and abrasion, respectively. The modified fouling index (FI) of flake-like particles within 31.5–40 mm is about 3.6 times that of regular particles within 50–63 mm. The shape indices of the ballast particles within 31.5–40 mm exhibit the most profound changes. The severities of the ballast breakage and fines generation (or modified FI) increased by 50% and 74%, respectively, due to the increase in the under-ballast support stiffness by 100 times and the drop height of 80 cm, respectively. The convexity and ballast breakage index (BBI) are promising for quantifying particle-degradation trends, and their statistical correlation found herein is potentially useful for the transition of ballast-bed-maintenance management from the current plan-based scheduling to condition-based upgrading.

## 1. Introduction

The ballast bed is among the main components of the ballasted track, of which the functionalities include transferring the wheel loads of moving trains, maintaining transverse and longitudinal track stability, facilitating track drainage, and protecting the track substructures underneath. Individual particles in the ballast bed constantly degrade in the form of abrasion, crushing (or breakage), or both under the repeated applications of dynamic train loading [1,2]. The detrimental degradation of ballast particles could directly deteriorate the in-service performance of ballasted tracks by altering the design gradation of the ballast bed, causing fouling and clogging problems, reducing particle interlocking and mechanical stability, and thus increasing differential track settlement [1,2,3]. Previous studies have shown that the degradation of aggregates can be greatly influenced by their gradation, morphology, and loading conditions [4,5,6,7]. In particular, the rapid development of both high-speed and heavy-haul railways in recent years has posed new challenges (i.e., the high frequency and/or high amplitude of train axle loads could potentially accelerate ballast degradation) [8]. The ballast beds on bridge decks were reported to degrade at a significantly higher rate than those on foundation soils due to the greater impact loads exerted on the former [9,10,11]. Such phenomena can be commonly observed in the embankment–bridge transition zones, where abrupt changes in the under-ballast support stiffness exist. Therefore, it is of great theoretical and practical importance to study the evolution of the ballast particle shape and crushing characteristics with repeated applications of the impact load under different in situ conditions.

A considerable number of studies, both discrete-element-method (DEM) simulations [12,13,14,15] and laboratory tests [16,17,18,19,20,21,22,23,24,25,26,27,28,29,30], have been conducted to reveal the degradation mechanisms of ballast materials. Liu et al. [14] investigated, from two-dimensional (2D) DEM simulations, the effect of the particle shape on the ballast abrasion characteristics, and they found that the lower sphericity values of the ballast particles led to greater Los Angeles abrasion (LAA) and a higher ballast breakage index (BBI). Ngamkhanong et al. [15] also studied the effect of the particle shape on the shear behavior of ballasts by using the DEM approach, and their results showed that the particle shape affected the formation of the shearing band. The DEM simulations provide great convenience to deepen the understanding towards the degradation mechanisms of railroad ballast materials; however, the micromechanical results obtained from these DEM simulations still lack direct and effective validation by laboratory test results.

The importance of the particle morphology on the hydromechanical performance of ballast materials has been long recognized. In recent years, both 2D and 3D imaging-based objective indices, in lieu of traditional subjective manual measurements, have been proposed to describe and quantify the morphological features, including the sphericity, angularity, flatness-and-elongation (F & E) ratio, roughness, and convexity [31,32,33,34,35,36,37]. However, few studies have focused on the comparative analysis of the changes in the 3D particle morphological descriptors before and after laboratory tests [20,21,23,28,29]. The existing laboratory tests for characterizing particle degradation mainly include the single-particle crushing test [16,17,18], direct shear test [19], LAA test [20,21,22,23], micro-Deval test [24], cyclic triaxial test [25,26], and impact test [27,28,29,30,31]. To characterize ballast particle degradation from the mesoscale perspective, researchers combined the 3D-laser-scanning and digital-image-processing techniques to quantify and compare the shape and surface roughness of ballast particles before and after laboratory tests [20,21,23,28,29]. Qian et al. [20] analyzed the morphological variations in the ballast particles with different numbers of turns during the LAA tests, and they studied the characteristics of abrasive degradation. They reported that there was a statistically significant correlation between the particle-shape indices and the fouling index (FI). Guo et al. [21] combined LAA tests with digital-image analysis and showed that ballast degradation is directly related to the particle size and shape, with flake- and needle-like ballast particles more susceptible to degradation. Bian et al. [23] quantified the particle-degradation process during the LAA tests by using morphological descriptors, and they showed that the angularity index (AI) and F & E ratio gradually decreased with the increasing number of drum turns, while the sphericity and convexity indices gradually increased. The comparison of the ballast-degradation trends observed in the field against those obtained from the LAA tests suggested that the commonly used LAA test may not truly reproduce the field-monitored changes in the particle size and shape properties [38]. Nimbalkar et al. [28] pointed out that ballast breakage caused by generated impact loads has been neglected in previous studies. Koohmishi et al. [29,30] conducted impact-load tests with different types of subgrades considered, and they combined them with digital image processing to study the changes in the morphology and breakage of ballast particles before and after tests. They reported that the flexible subgrade greatly slowed down the rate of the reduction in the angularity and surface-roughness indices. Even though the macroscopic mechanical behavior of ballast materials has been well studied so far, there is still a lack of studies on the evolution law of the mesoscale degradation characteristics, including the particle size, shape, and breakage under impact loading.

In track transition zones, and other locations where rail irregularities are commonly exasperated, the ballast bed is often subjected to extra considerable impact loads that increase with the increasing train speed [39]. This could potentially aggravate the performance degradation and thus compromise the operational safety and riding comfort [40,41,42]. Hence, it is important to study how the crushing characteristics and shape and size properties of ballast particles evolve with the repeated applications of such high-impact loading. Qian et al. [43] employed an imaging-aided DEM modeling methodology to investigate the track deformation behavior of four different ballast materials with varying combinations of gradation and shape properties. Their results showed that the proper selection of the gradation and shape properties of ballast materials can effectively reduce the settlement in the ballasted-track transition zone. Nevertheless, few studies have addressed the influence of varying particle sizes and shape properties, or the underlying foundation stiffness.

According to Indraratna et al. [44], the field-measured maximum amplitude of the vertical cyclic stress underneath the tie plates is approximately 230 kPa, as generated by an axle load of 25 tons traveling at 60 km/h. Koohmishi et al. [30] measured the impact forces generated by a hammer of 50 kg free falling from a height of 42 cm above the top surfaces of ballast specimens underlain by different types of subgrade materials. They reported that the impact force of 3.5 tons (or equivalently, vertical compressive stress of 780 kPa) was generated on the top surface of the ballast specimens over a rigid subgrade, whereas the vertical compressive stress was 450 kPa for a flexible subgrade. It becomes clear that the abovementioned two studies reported similar orders of magnitude of vertical compressive stress. Therefore, the use of impact-load tests to simulate the moving train loading-induced impact loading exerted on typical ballast beds can be reasonably justified. It is worth mentioning that the impact load applied in the laboratory tests of this study refers to and simulates the dynamic vertical wheel load-induced impact loading transmitted to the top surface of the ballast bed, rather than the difference between the dynamic and static vertical wheel loads.

In this study, laboratory impact tests were deliberately designed to address the important factors that influence ballast particle degradation, including the particle size and shape properties, the magnitude of the impact load, the number of impact-load applications, and the underlying support stiffness. A falling-weight apparatus was customized and employed for this purpose. The unbound ballast specimens were prepared and loaded according to the designed testing matrix. At different stages of each impact-loading test, the changes in the ballast particle size and shape properties were quantified by mechanical-sieve analysis and digital-image analysis. The morphology and breakage evolution characteristics of railroad ballasts under different engineering circumstances were studied and revealed.

## 2. Materials, Testing Program, and Methods

### 2.1. Materials Tested

**Figure 1:**
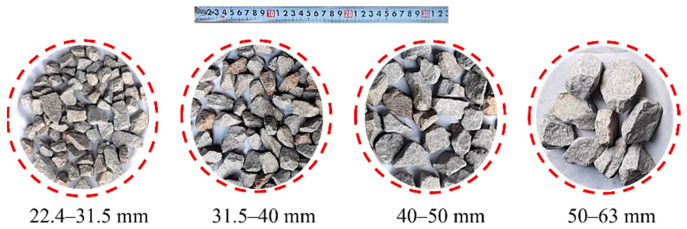
Illustration of the ballast particles within four different size ranges.

The ballast materials tested in this study were sampled from a commercial quarry of crushed stones in Xiangxiang, Hunan province, China. The lithology of sharp-edged ballast particles is hard dense granite. To avoid the interference from the dust and mud sticking to the surfaces of the ballast particles in the particle-morphology acquisition and impact-load-test results, the ballast materials were cleaned prior to the laboratory tests and then air-dried thoroughly. They were then sieved into four different size groups of 22.4–31.5 mm, 31.5–40 mm, 40–50 mm, and 50–63 mm, as illustrated in Figure 1.

### 2.2. Description of Laboratory Testing Program

#### 2.2.1. Impact-Loading Apparatus

**Figure 2:**
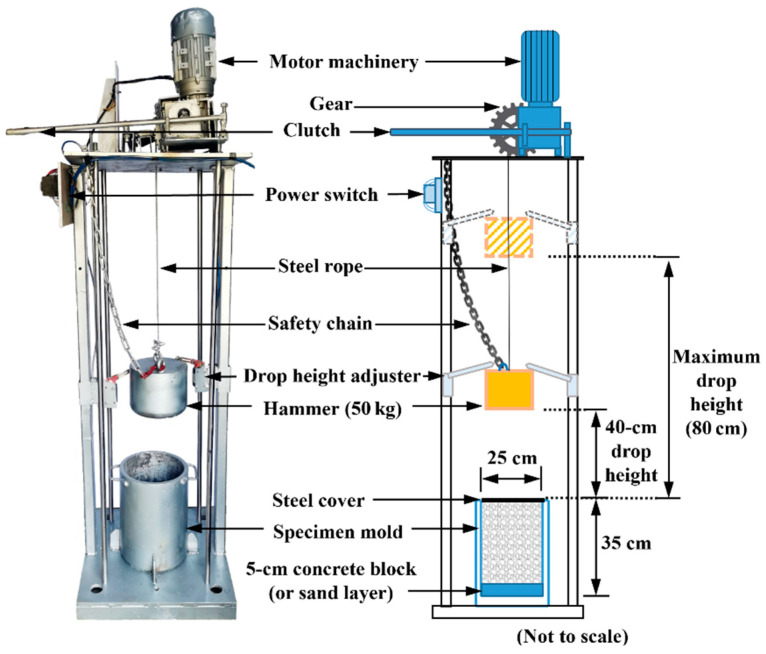
Illustration of the setup and configuration of the customized impact-load-test apparatus.

The apparatus used for the impact-load tests was customized particularly for this study, and the entire setup and configuration are shown in Figure 2. It consists of an electrical motor system, lifting module, stopping module, free-fall hammer, loading frame, and specimen mold. The electrical motor drives the gears to roll up the hammer to the prescribed height of interest via the wire rope attached to it, whereas the stopping module (drop-height adjuster), once triggered, can automatically shut off the motor and gear system upon emergency events or the completion of the test. The hammer falling freely at a specified height can apply an impact load of a certain magnitude to the ballast specimen.

The steel hammer weighs 50 kg and measures 20 cm in diameter, while the maximum drop height is 80 cm, which measures from the bottom surface of the hammer to the top surface of the metal mold. Note that the vertical stress levels generated by the impact load on the top surface of the ballast specimens were not explicitly measured in this study; nevertheless, they were expected to approximate those reported by Koohmishi et al. [30], as the weight and free-fall height of the hammer were nearly identical in both studies. On the basis of the laboratory-measured results by Koohmishi et al. [30], the amplitude of the vertical compressive stress generated by the free drop of the 50 kg hammer from a height of 42 cm is estimated to be around 780 kPa and 450 kPa at the top surface of the ballast specimen over rigid and flexible supports, respectively. The unbound ballast specimens, fabricated according to the testing matrix, were placed into the metal mold, of which the inner diameter and the height are 25 cm and 35 cm, respectively. To simulate different types of foundations underneath the ballast bed, a cylindrical concrete block and a sand layer were placed at the bottom of the mold, both of which were 5 cm in thickness. The height of each of the ballast specimens was 30 cm, thus resulting in a bulk volume of 14,726.2 cm^3^ and an approximate weight of 15 kg. The mold was fixed to the base plate of the impact-loading apparatus in order to prevent it from moving during the test. A steel cover of a 25 cm diameter was placed on the top of each ballast specimen to avoid stress concentration, and to ensure the uniform distribution of the impact load generated by the free-falling hammer. It is worth mentioning that the ratio of the ballast-specimen diameter to the maximum ballast particle size was around 4.0 in this study, which is still within the range of 3.84–4.8 reported in previous similar studies [29,30], as well as within the range of 3.74–4.4 specified by European and Chinese standards [45,46]. Hence, the interference of the size and boundary effects on the laboratory impact-load-test results can be minimized reasonably well, although a specimen diameter of at least 300 mm is recommended for future laboratory tests of ballast materials.

**Figure 3:**
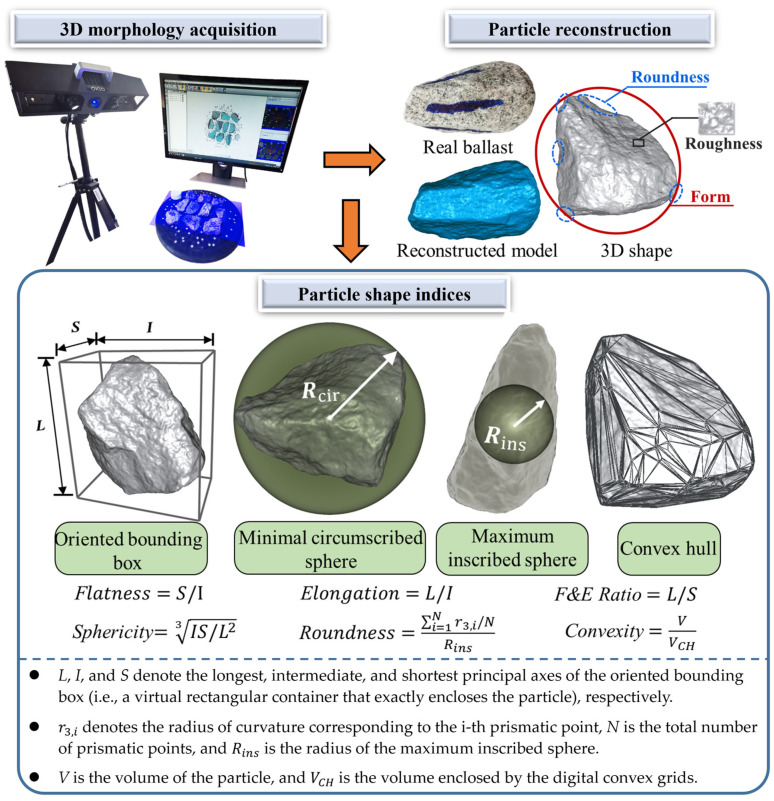
Illustration of reconstructing digital 3D models of selected ballast particles and calculating their shape indices.

To characterize the evolution of the ballast particle morphology and size properties with the number of impact applications, a 3D laser scanner was used to reconstruct 3D models of the ballast particles selected, and then calculate their morphological indices, of which the flowchart procedures are illustrated in Figure 3. The laser scanner used is equipped with a high-precision industrial camera, with the measurement accuracy ranging from 0.015 mm to 0.035 mm, and it adopts blue-light narrow-band filtering technology to effectively reduce the interference of external ambient light. The selected ballast particles were first placed onto a rotary base and were then exposed to the blue gratings sent by the 3D scanner (see Figure 3) to generate the point clouds and triangular surface grids. With the aid of 3D-image-processing techniques, the 3D digital models of those ballast particles were reconstructed accordingly. Subsequently, a selection of morphological descriptors of each particle was calculated from its reconstructed 3D model via the self-written Matlab^®^ codes (i.e., the flatness-and-elongation ratio (F & E ratio), sphericity, roundness, and convexity, as shown in Figure 3. Additional details of such shape indices, including the calculation formulas, are described elsewhere [32,33,34,35,36]).

#### 2.2.2. Impact-Load Tests for Single-Size Ballasts

To investigate the effects of the particle size, shape, and number of impact applications on the ballast particle degradation, a series of laboratory impact-load tests were conducted first on single-size-ballast samples. According to local Chinese standard (TB/T2140.2–2018) [46], four different particle size intervals (i.e., 22.4–31.5 mm, 31.5–40 mm, 40–50 mm, and 50–63 mm) were selected for use in this study. Note that the term “single size ballast” hereinafter refers to the ballast assembly that has the sizes of particles that mainly belong to a single sieve size. For instance, a 21.5–31.5 mm single-size ballast represents the assembly of ballast particles of which most pass a 31.5 mm sieve but are retained on the immediate smaller sieve (i.e., 22.4 mm). Based on the calculated flatness-and-elongation parameters (see Figure 3) [46,47,48], the original ballast particles were classified into three different shape categories prior to any impact-load tests: a flake-like shape for a flatness less than 0.6, a needle-like shape for an elongation greater than 1.8, and a regular shape for all the rest [46].

**Figure 4:**
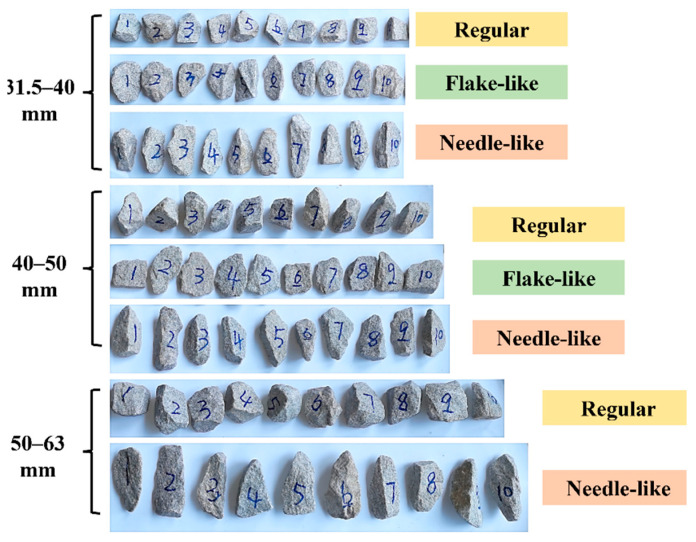
Illustration of example ballast particles of different shape categories and size ranges.

Note that a few needle-like or flake-like particles existed within the size range of 22.4–31.5 mm for the ballast samples collected from the quarry; thus, this particular size range was excluded from the test matrix for single-size ballasts. In addition, a few flake-like particles existed within the size range of 50–63 mm; thus, only two shape categories were studied for this specific size range (i.e., regular and needle-like). Figure 4 exemplifies the ballast particles selected from different shape categories for different size ranges (i.e., 10 particles numbered from 1 to 10 for each combination of shape and size range). The initial weight of each cylindrical ballast specimen was 15 kg. Upon the completion of every 10 impact-load applications, the ballast specimen was re-sieved and re-weighed. The fixed free-fall height of 60 cm was set consistently. The fixed free-fall height of 60 cm was adopted in this study to apply higher impact energy than that of 40 cm to the single-size ballasts of varying particle-shape categories. The goal was to study the relative importance of individual ballast particle size ranges on the severity of the particle abrasion and breakage under the same level of impact energy. The fixed free-fall height of 60 cm was expected to simulate the impact loading generated by a moving train experienced in the field reasonably well [49]. The specific impact-load-test matrix for single-size ballasts is summarized in Table 1.

#### 2.2.3. Impact-Load Tests for Multiple-Size Ballasts

**Figure 5:**
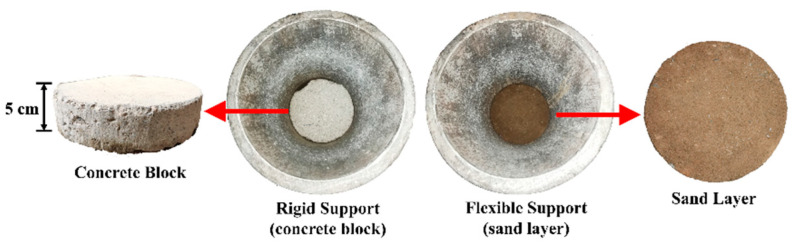
Illustration of the concrete block and sand layer used to represent rigid and soft under-ballast support, respectively.

**Figure 6:**
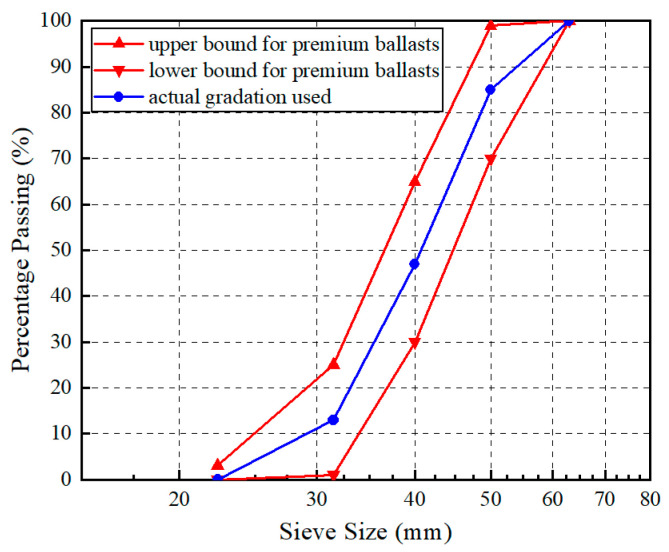
The particle-size-distribution curves of ballast materials designed for impact-load tests.

The existing field observations discovered that the ballast beds over rigid bridge decks often experience more severe particle breakage than those over flexible embankments [9]. To comparatively explore the changes in the particle size, shape, and breakage properties of ballast specimens underlain by both rigid and flexible supports, the 5 cm-thick concrete block and the 5 cm-thick sand layer were placed at the bottom of the molds, respectively, as sketched in Figure 5. The typical resilient-modulus (M_r_) value of regular Portland cement concrete materials is about 31 GPa, whereas the typical M_r_ value of regular unbound granular embankment fill materials (including the sand-layer material in this study) is about 300 MPa [50]. Hence, the rigid concrete block is approximately 100 times stiffer than the soft (flexible) sand layer in this study. It should be noted that the geotextile was placed in between the ballast and sand materials to physically separate them, and to refrain the ballast particles from infiltrating into the sand layer. Four different drop heights of the impact hammer (i.e., 20, 40, 60, and 80 cm) were prescribed to study the effect of the impact-load magnitude on the changes in the ballast particle size and breakage characteristics. In fact, the two different drop-height levels of 29.6 cm and 42 cm are prescribed for impact tests in the European standards. According to the actual measurements reported by Koohmishi et al. [30], the vertical stress levels caused by the 50 kg hammer falling from a height of 42 cm are 780 kPa and 450 kPa on the top surface of ballast specimens over rigid and flexible supports, respectively. In addition, as reported by Indraratna et al. [44], who conducted a field instrumentation study on a ballasted railway track, the peak vertical stress of 415 kPa was recorded under the passage of the coal-hauling train with an axle load of 25 tons. In this study, four different drop-height levels (20, 40, 60, and 80 cm) were adopted. Specifically, the drop-height levels of 20 cm and 40 cm were meant to simulate the existing regular dynamic vertical wheel load levels, while the drop-height levels of 60 cm and 80 cm were intended to accommodate higher dynamic vertical wheel load levels due to the increasingly heavier axle load and faster travel speed, and especially at locations where wheel/rail irregularities, rail joints, and sleeper–ballast gaps, among others, commonly exist. The ballast gradation for the impact-load tests in this stage was designed according to the Chinese standard (TB/T2140.2-2018) [46] (Figure 6), and the ballast specimens tested were prepared by following the design gradation. Note that the contents of the needle- and flake-like particles in these ballast specimens were intentionally controlled within 10%, as required by the local standard [46].

The specific impact-load-test matrix for multiple-size ballasts is shown in Table 2. Note that the particle-shape quantification was conducted for ballast specimens loaded by the hammer dropping from the height of 60 cm, while the other ballast specimens were not subjected to particle-shape quantification due to the limitation of the overwhelming testing workload. Specifically, prior to the impact-load tests, all particles within the size ranges of 40–50 and 50–63 mm, and approximately 30 particles within each of the two size ranges (i.e., 22.4–31.5 and 31.5–40 mm), were randomly handpicked from the related ballast specimens, labeled with a marker pen, and then laser-scanned for 3D model reconstruction and the shape-index calculation. The process of particle-shape quantification was repeated every 20 impact-load applications to track the evolution of the particle size, shape, and breakage with the number of impact-load applications.

### 2.3. Particle-Breakge Quantification

**Figure 7:**
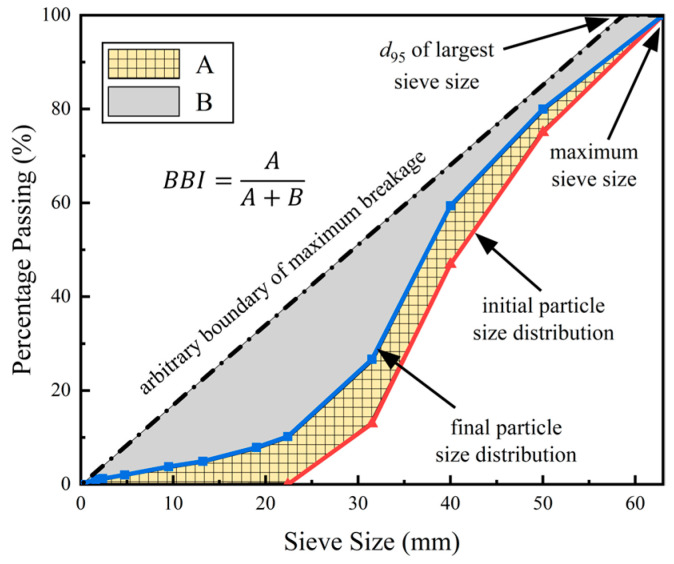
Illustration of the definition of ballast breakage index (BBI).

To further quantify and analyze the ballast particle breakage, four commonly used particle-breakage indices were calculated for different numbers of impact-load applications. Marsal [4] proposed the breakage index ($B_{g}$) in 1967, which is defined as the sum of the positive value of the difference in the percentage content of each sieve size before and after particle breakage ($\Delta W_{k}$) (see Equation (1)). The particle-breakage index ($B_{g}$) is simple and straightforward to calculate, and it can better measure the overall fragmentation of ballast specimens; however, the $B_{g}$ is influenced by the sizes of individual particles and the gradation of the entire specimen, which thus makes it difficult to quantify the actual changes in the individual particle size ranges. Indraratna [25] proposed a breakage index (BBI) for ballast particles, which is defined as the ratio of the area enclosed by the post-test and initial gradation curves to the area enclosed by the initial and ultimate gradation curves (see Equation (2)). They reported that ballast particle degradation causes the initial particle size distribution to shift towards finer gradations (i.e., the fraction of smaller particle sizes increases), while the fractions of larger particle sizes exhibit insignificant changes due to impact loading. Therefore, instead of defining the breakage potential according to the changes in individual particle sizes, they proposed the use of the ultimate breakage boundary, which actually seems more appropriate. The BBI index can reflect the combined breakage of different particle sizes more completely, and its calculation scheme is shown in Figure 7 (where A denotes the area enclosed by the initial and final gradation curves, and B denotes the area enclosed by the final and ultimate gradation curves). The fractal dimension (D) derived from the Tyler model [51] is another index for quantifying particle breakage. The fractal theory is mainly used to characterize a variety of complex and discontinuous shapes and processes, of which the core features remain unchanged in nature as the system is scaled up or down. The fractal grading equation is simple in its form, with only one single parameter involved, and thus it has been widely used to study the gradation evolution of stone-based materials. It can be calculated from Equations (3)–(4), where $M_{\left(\rho <d_{i}\right)}$ is the cumulative mass of particles smaller than $d_{i}$;$M_{T}$ is the total mass of particles; $M_{\left(\rho <d_{i}\right)}$/$M_{T}$ is the percentage by mass of particles smaller than $d_{i}$; $d_{max}$ is the maximum particle size; *α* is the slope of the fitted linear line between $M_{\left(\rho <d_{i}\right)}$/$M_{T}$ and $d_{i}$/$d_{max}$ in logarithmic scales (i.e., the log–log plot). Selig et al. [3] proposed the fouling index (FI) to quantify the influence of fine fractions in the ballast bed, which is defined as the sum of $P_{4.75}$ (percent mass of materials passing 4.75 mm sieve) and $P_{0.075}$ (percent mass of materials passing 0.075 mm sieve). During the laboratory tests, the 0.075 mm sieve was broken and then discarded for use due to the shortage of replacement sieves at that time (amid the COVID-19 outbreak in Changsha, China). Therefore, the smallest sieve size of 1.7 mm was alternatively used in the calculation of the classical fouling index to substitute for the original 0.075 mm sieve size. The original FI was modified in this study as the sum of $P_{4.75}$ and $P_{1.7}$ (percentage by mass of materials passing 1.7 mm sieve), as shown in Equation (5):

(1) $$B_{g}=\sum \Delta W_{k}$$

(2) $$BBI=\frac{A}{A+B}$$

(3) $$\mathrm{lg}\left[\frac{M_{\left(\rho <d_{i}\right)}}{M_{T}}\right]=\left(3-D\right)\mathrm{lg}\left(d_{i}/d_{max}\right)$$

(4) D=3−α

(5) $$FI=P_{1.7}+P_{4.75}$$

### 2.4. Particle-Shape Quantification

Ballast particle morphology is one of the important factors affecting the mechanical stability of the ballast bed and railway track. The particle-scale shape can be described from three different aspects: form, angularity (or roundness), and surface roughness [48]. In order to quantify the changes in the particle shape and disclose the shape-related ballast-degradation trends during the applications of the impact load, the selected ballast particles of interest were subjected to 3D laser scanning, with their digital 3D models reconstructed accordingly. The basic geometrical dimensions of these reconstructed 3D particle models, including the long axis (*L*), medium axis (*I*), short axis (*S*), volume (*V*), and convex hull (*CH*), were calculated, from which the commonly used shape indices were further calculated according to Figure 3. The entire process is also illustrated as flowcharts in Figure 3.

To be specific, the flatness-and-elongation ratio (F & E ratio) index describes the overall form of a particle, with its value being 1 for perfect spheres, and with greater values representing slenderer particles. The sphericity index measures the similarity among the length, width, and height dimensions of a particle, and it thus describes the resemblance of the particle to the ideal sphere (of which the sphericity value is 1). The roundness index is defined as the ratio of the radius of curvature at the sharp corners of the particle boundary to the overall size of the particle, and it thus describes the sharpness of all the corners of the particle. The convexity index describes the concaveness of a particle, with a value closer to 1 representing a lower severity of concaveness. More details of such shape indices and their applications can be found elsewhere for brevity [32,33,34,35,36].

Prior to conducting the impact-load tests for multiple-size ballasts under the combinations of a 60 cm drop height and two different types of under-ballast supports, a statistically sufficient number of ballast particles were sampled from all the size ranges of the initial specimens, numbered by a marker pen, and then laser-scanned for digital 3D model reconstruction. This process was repeated upon the completion of every 20 impact-load applications to track and quantify the particle-shape-degradation trends. The ballast particles of the specimens tested were subjected to 3D laser scanning prior to and upon the completion of a prescribed number of impact-load applications, from which the rate of mass reduction (η) can be calculated via Equation (6):

(6) $$\eta =\frac{V_{1}-V_{2}}{V_{1}}$$

where $V_{1}$ denotes the initial volume of a ballast particle scanned before the impact-load test, and $V_{2}$ denotes the volume of a degraded ballast particle scanned upon the completion of a prescribed number of impact-load applications.

## 3. Testing Results and Analysis

### 3.1. Impact-Load-Test Results of Single-Size Ballasts

**Figure 8:**
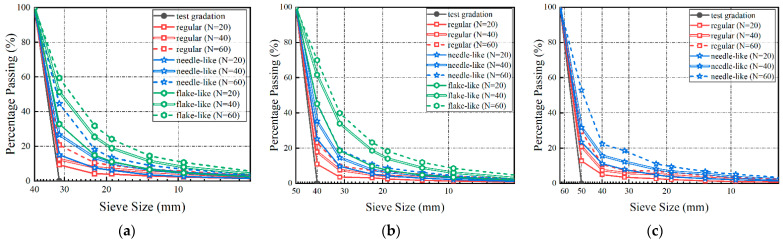
Evolution of particle size distributions with the prescribed number of impact-load applications (N) for distinctly shaped single-size ballasts: (**a**) 31.5–40 mm; (**b**) 40–50 mm; (**c**) 50–63 mm.

Figure 8 shows the variations in the particle size distribution of the initial single-sized ballast specimens with the number of impact-load applications. To ease the observation and comparison of the variation trends, only the resulting gradation curves measured from mechanical-sieve analysis upon the 20th, 40th, and 60th impact-load applications are plotted in Figure 8. It can be seen that the ballast particles within each of the three different single-size groups experienced profound particle breakage. That is, as the number of impact-load applications (N) increases, the gradation curves move towards the upper-right direction, the content of finer particles increases, and the particles gradually become better packed. It can also be observed from Figure 8 that the initial particle shape has a significant effect on the ballast particle breakage, with flake-like particles being the most crushable, followed in descending order by the needle-like and regular-shaped particles.

**Figure 9:**
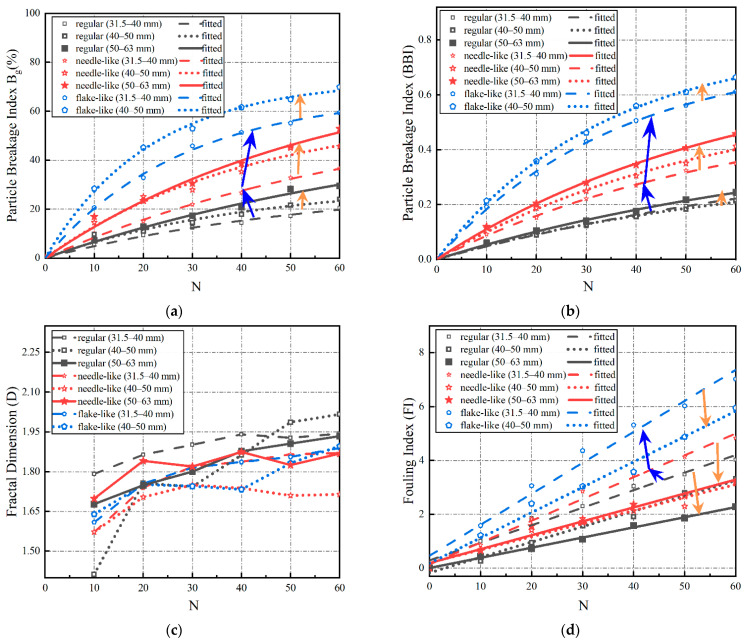
Evolution of four particle-breakage indices with the number of impact-load applications (N) for distinctly shaped single-size ballast specimens: (**a**) $B_{g}$; (**b**) BBI; (**c**) D; (**d**) modified FI.

The abovementioned four breakage indices were calculated for each single-size ballast specimen upon different numbers of impact-load applications, as shown in Figure 9 (where the blue and orange arrows illustrate the trends of variations). It can be seen from Figure 9 that, as the number of impact-load applications increases, the three indices, $B_{g}$, BBI, and modified FI, all exhibit clear ascending trends, while the fractal-dimension (D) index exhibits fluctuating trends. This may indicate that the fractal dimension (D) is not suitable for describing and quantifying the particle breakage evolution of single-size ballasts. As shown in Figure 9a,b, the $B_{g}$ and BBI indices exhibit similar ascending trends with an increasing number of impact-load applications (N), and the rate of increase of the $B_{g}$ and BBI gradually increases for N < 30, and then starts to decrease for N > 30. Therefore, Equation (7) was proposed to statistically relate the $B_{g}$ and BBI with the N, and the fitted coefficients are listed in Table 3. The limit values of the $B_{g}$ and BBI indices of each single-size ballast can thus be obtained by solving the limit of Equation (7) accordingly. As indicated by Equation (8), such limits of the $B_{g}$ and BBI indices are equal to the fitted values of the coefficient a (b is a positive number). By examining Figure 9a,b and the fitted values of the coefficient a (see Equation (8)) together, it can be seen that both the particle shape and size affect ballast particle breakage, with the particle shape being relatively more influential. The larger particle size results in a greater ultimate breakage limit, and more profound breakage phenomena. The ultimate breakage limit of the single-size ballast of a regular shape is the smallest, followed in ascending order by those of the single-size ballasts of needle- and flake-like shapes. Quantitatively, the breakage of the flake-like particles is about 1.5–1.66 times and 1.25–1.5 times higher than that of the regular and needle-like particles, respectively.

**Figure 10:**
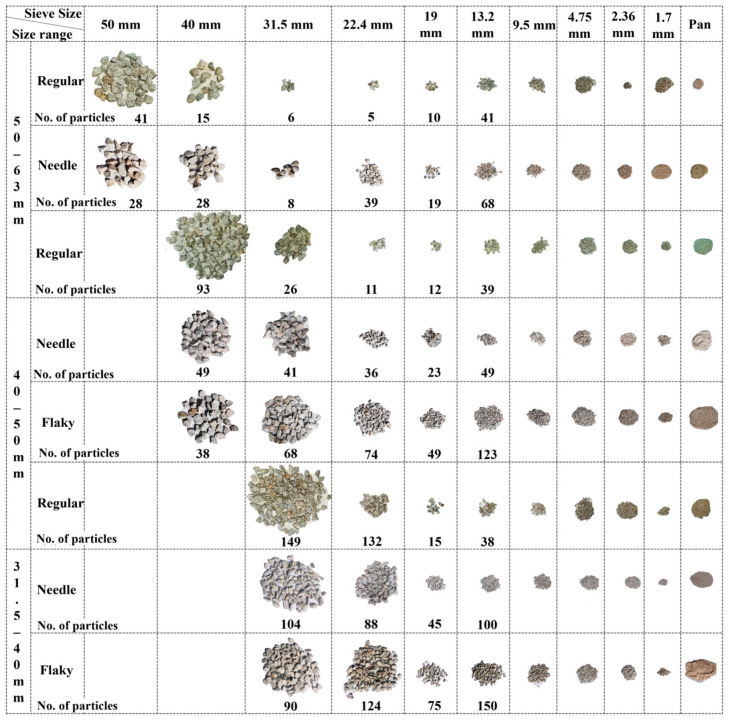
Photos of particle-size-degradation trends of distinctly shaped single-size ballasts upon the completion of 60 impact-load applications.

It can be seen from Figure 9d that the modified fouling index (FI) increases approximately linearly with the increasing number of impact-load applications (N); therefore, Equation (9) was proposed to fit this linear relation statistically, and the fitted coefficients for different single-size ballasts are listed in Table 3. The slope (*k*) in Equation (9) denotes the rate of the modified FI increase with the number of impact-load applications, and the greater *k* value indicates a higher content of fine particles in the ballast specimen. It can be seen from Figure 9d and Table 3 that the flake-like ballasts yield the greatest slope (k) value, followed in descending order by the needle-like and regular-shape ballasts. This implies that flake-like ballasts generate more fines during the degradation process. In addition, the slope (*k*) value of the large single-size ballasts is lower than that of the small single-size ballasts, indicating that the former yields less fines than the latter during impact-load tests. The modified fouling index (FI) of flake-like particles within the size range of 31.5–40 mm is about 3.6 times that of regular particles within the size range of 50–63 mm. By comparing the variations in the $B_{g}$ and BBI indices for single-size ballasts with different combinations of particle sizes and shapes, it can be concluded that, under impact loading, large single-size ballasts degrade mainly by breakage, whereas small single-size ballasts degrade mainly by abrasion. This conclusion can also be further visualized in Figure 10. Distinctly shaped single-size ballasts were sieved into different size groups upon the completion of 60 impact-load applications, and they were then photographed to visually compare the particle-size-degradation trends. Note that the photos illustrating the crushing process of individual ballast particles of different shape categories, including flake-like ones, were not captured during the laboratory impact tests, which is partly due to the challenges of tracking child particles fragmented from individual parent particles (which were assembled in the ballast specimens). However, such important information deserves to be attempted in future studies.

(7) $$y=a*\left(1-e^{-bx}\right)$$

(8) $$\underset{x\to \infty }{\lim}y=\underset{x\to \infty }{\lim}a*\left(1-e^{-bx}\right)=a$$

(9) y=a+kx

### 3.2. Impact-Test Results of Multiple-Size Ballasts

**Figure 11:**
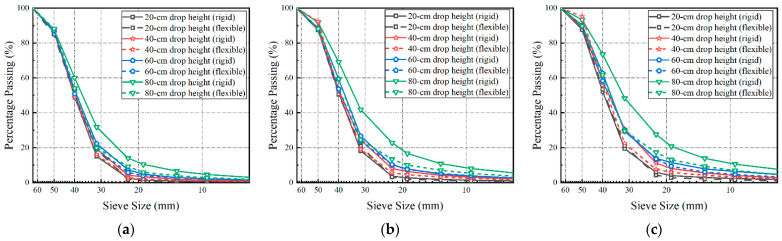
Evolution of the gradation curve of each multiple-size ballast specimen with different numbers of impact-load applications (N): (**a**) N = 20; (**b**) N = 40; (**c**) N = 60.

In the impact-load-test matrix for multiple-size (full gradation) ballasts, two different under-ballast supporting conditions (i.e., a rigid concrete block versus soft sand layer) and four different drop heights (i.e., 20, 40, 60, and 80 cm) were added, as summarized in Table 2. Figure 11 shows the variations in the gradation curve of each multiple-size ballast specimen, with prescribed numbers of impact-load applications (i.e., 20, 40, and 60), under different testing conditions. As can be seen from Figure 11, with the same number of impact-load applications (N) and the same drop height (H), the gradation curve of each multiple-size ballast specimen underlain by the rigid concrete block moves towards the upper-right direction more dramatically than that of the specimens underlain by the soft sand layer. This indicates that the rigid under-ballast support could exacerbate particle breakage as compared with the soft support. As the drop height or the number of impact-load applications gradually increases, the severity of the ballast particle breakage gradually increases, regardless of the under-ballast support stiffness. The most severe particle breakage was induced by the combination of an 80 cm drop height and 60 impact-load applications.

**Figure 12:**
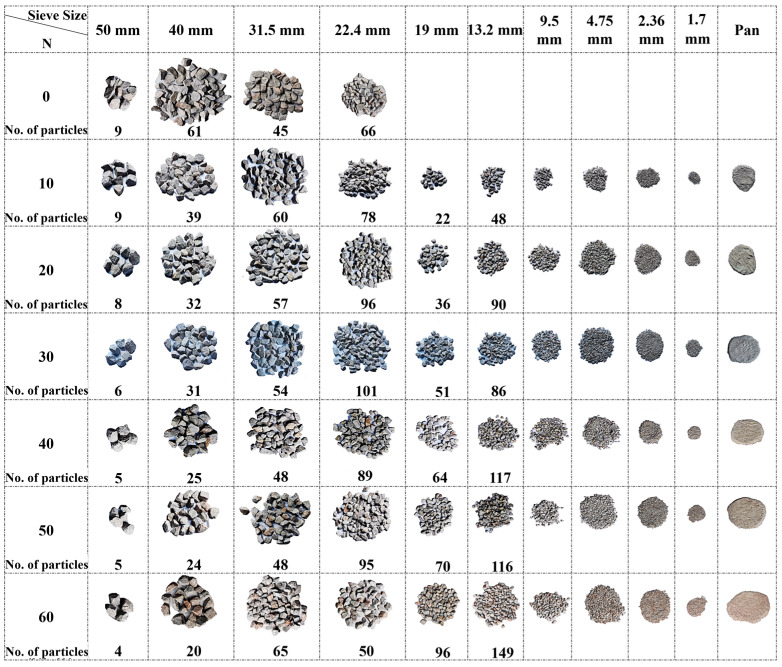
Photos of particle-size-degradation trends of regularly shaped multiple-size ballasts upon the completion of every 10 impact-load applications under the combination of a rigid concrete block support and an 80 cm drop height.

Figure 12 shows the photos of the particle-size-degradation trends of multiple-size ballast specimens with every 10 impact-load applications under the combination of a rigid concrete block support and an 80 cm drop height. As can be seen from Figure 12, the main degradation pattern of the multiple-size ballast specimen is the breakage of large particles (40~50 cm in size) during the early stage of impact loading (N < 20), and it then transits to the breakage and pulverization of fine particles for N > 20 (when the changes in the particle size are insignificant for large particles). This may imply that, as the ballast bed gradually degrades, the generated fine particles gradually fill the pores formed by larger particles in contact, and they serve to buffer the stress concentrations and thus protect the larger particles. From this stage onward, the degradation of ballast particles is mainly attributable to the breakage and pulverization of fine particles.

**Figure 13:**
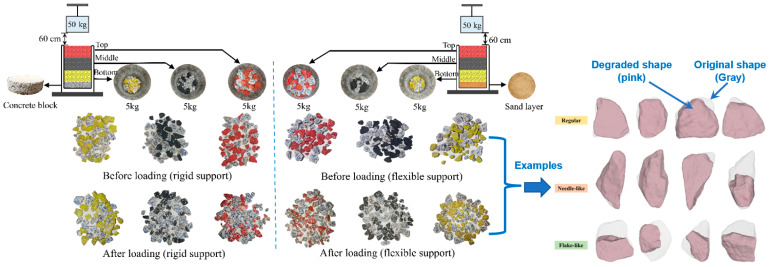
Photos of particle abrasion and crushing trends among different layers of multiple-size ballast specimens supported by a rigid concrete block and relatively soft sand layer under a 60 cm drop height.

To visualize and further compare the particle abrasion and crushing trends at different depths of the ballast specimens supported by a rigid concrete block and a relatively soft sand layer, respectively, each initial specimen was divided into top, middle, and bottom layers, and the particles located in these layers were painted red, black, and yellow, respectively, prior to the impact-load tests. The drop height was set as 60 cm, as shown in Figure 13. The new particles subsequently generated upon the completion of different numbers of impact-load applications were marked by numbers without painting. To comparatively analyze the particle-shape-degradation trends among these three different layers of multiple-size ballast specimens supported by a rigid concrete block and a relatively soft sand layer, Figure 13 shows the photos of the particles located in different layers prior to the impact-load tests, and upon the completion of 60 impact-load applications. On the one hand, by examining the disintegrated painted particles, it was found that the ballast particles supported by the rigid concrete block were more severely crushed and abraded as compared with those supported by the relatively soft sand layer. On the other hand, the particles in the top experienced the most severe breakage and abrasion, followed in descending order by those in the middle and bottom layers. In particular, negligible breakage or abrasion was observed for the particles in the bottom layer.

**Figure 14:**
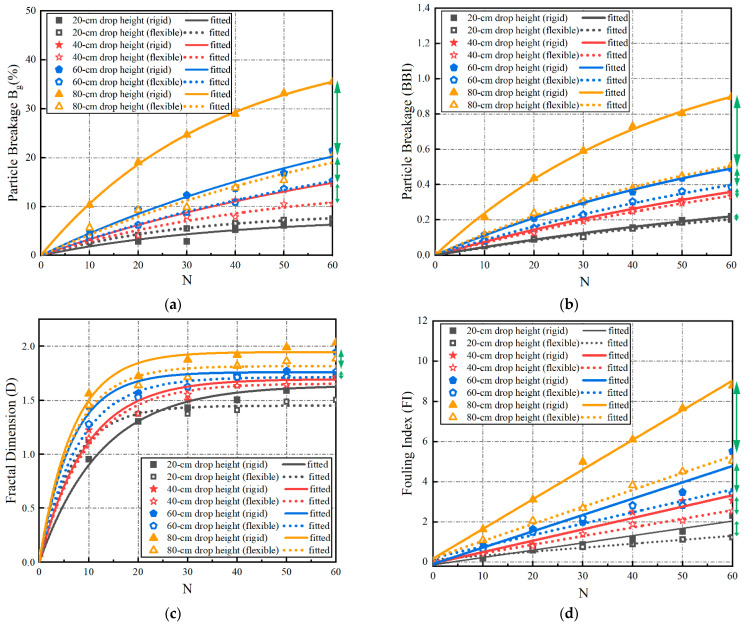
Evolution of four particle-breakage indices with the number of impact-load applications (N) for multiple-size ballasts under different combinations of under-ballast supports and drop heights: (**a**) $B_{g}$; (**b**) BBI; (**c**) D; (**d**) modified FI.

The evolution of the particle breakage of the multiple-size (or full gradation) ballast specimens with the number of impact-load applications was quantified by the aforementioned $B_{g}$, BBI, fractal dimension (D), and modified fouling index (FI), with the results plotted in Figure 14 (where the lengths of green arrows indicate the differences in particle breakage between rigid and flexible under-ballast supports under the same drop height of the hammer). The statistical correlations among the ballast particle breakage, under-ballast support stiffness, drop height of the hammer, and number of impact-load applications were further explored. As illustrated in Figure 14, the breakage indices (the $B_{g}$, BBI, D, and modified FI) all increase clearly with the increasing number of impact-load applications (N). From Figure 14a,b, it can be seen that the variations in the $B_{g}$ and BBI with the N share great similarity. Hence, the previously proposed Equation (7) was employed once again to fit the statistical correlations existing between the $B_{g}$ (or BBI) and N, and the fitted coefficient values are summarized in Table 4. The limits of the $B_{g}$ and BBI were then calculated as per Equation (8). By examining Figure 14a,b and Table 4 together, it can be concluded that the particle breakage of the multiple-size ballast specimens supported by the rigid concrete block was more severe than that of the specimens supported by the flexible sand layer, and especially for a higher drop height (or equivalently, a higher impact load). With the gradual increase in the drop height, the differences in the particle breakage between the rigid and flexible under-ballast supports increase moderately as well. As for the drop height of 80 cm, the particle breakage ($B_{g}$ or BBI) could increase by 50% due to the increase in the under-ballast support stiffness by 100 times. Therefore, it can be interpreted that, as the dynamic wheel load exerted by moving trains increases due to an elevated axle load and/or running speed, the ballast bed underlain by a rigid support (e.g., bridge decks) would be more prone to breakage than that underlain by a soft support (e.g., embankment soils). From this perspective, it becomes imperative to conduct in-depth studies on the degradation mechanisms of ballast beds over bridge decks, and to then recommend proper technical countermeasures to prevent, mitigate, or rectify such degradation.

From Figure 14c, it can be seen that the growth rate of the fractal dimension (D) of each multiple-size ballast specimen gradually attenuates as the number of impact-load applications increases (i.e., the growth rate of D increases for N < 20, and decreases for N > 20, gradually until plateauing). Equation (7) was attempted to statistically relate the fractal dimension (D) with the number of impact-load applications (N), and the limits (i.e., the coefficient a) of the fractal dimension (D) of different multiple-size ballast specimens were solved accordingly and are summarized in Table 4. By examining both Figure 14c and Table 4 together, it can be found that the fractal dimension (D) increases with the increasing drop height of the free-falling hammer. From Figure 14d, it can be seen that the modified fouling index (FI) of each multiple-size ballast specimen demonstrates a linearly ascending trend with the increasing number of impact-load applications (N); hence, Equation (9) was used herein to express the relation between the modified FI and the N, instead of Equation (7). Table 4 indicates that, as the drop height gradually increases, the slope (k) of the modified fouling index (FI) gradually increases (i.e., the abrasion and pulverization of ballast particles are consequently exacerbated). When the drop height was 80 cm, the modified FI value for the rigid under-ballast support was about 1.72 times greater than that for the flexible support (i.e., the amount of fine-particle generation increases by 74%, thus indicating that the existence of a rigid support aggravates not only the particle crushing, but also the interparticle abrasion of the ballast bed, and particularly under higher levels of impact load.

**Figure 15:**
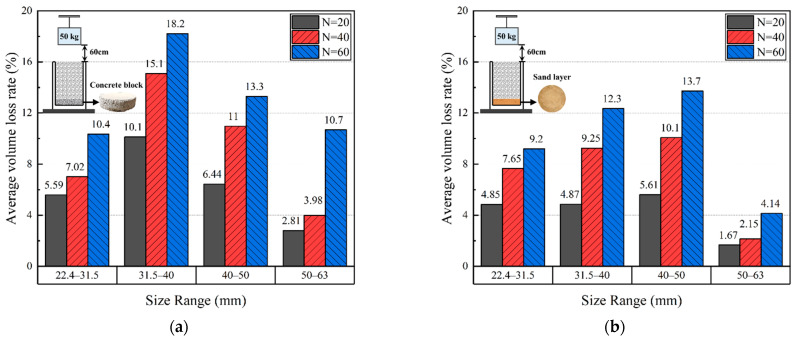
The histograms of average rates of volume reduction (η) of particles within different size ranges of the multiple-size ballast specimens over (**a**) rigid and (**b**) flexible supports upon the completion of 20, 40, and 60 impact-load applications (N).

Figure 15 shows the histograms of the average rates of the volume reduction (η) of particles within each size range of the multiple-size ballast specimens over rigid and flexible under-ballast supports upon the completion of 20, 40, and 60 impact-load applications. Overall, the average rate of the volume reduction (η) corresponding to the rigid under-ballast support is greater than that corresponding to the flexible under-ballast support. This further substantiates the previous findings that the ballast bed underlain by rigid support is subjected to greater stress levels and is thus likely to experience more severe particle breakage and abrasion than that underlain by soft support. Under the identical testing condition, the average rate of the volume reduction (η) is the lowest for the largest ballast particles (50–63 mm in size), and the highest for the intermediate ballast particles (31.5–40 and 40–50 mm in size), with the middle values taken by the smallest ballast particles (22.4–31.5 mm in size). This observation may be explained by the fact that larger particles possess greater surface areas and thus have larger contact areas with other neighboring particles, which leads to less stress concentration and volume reduction. 

**Figure 16:**
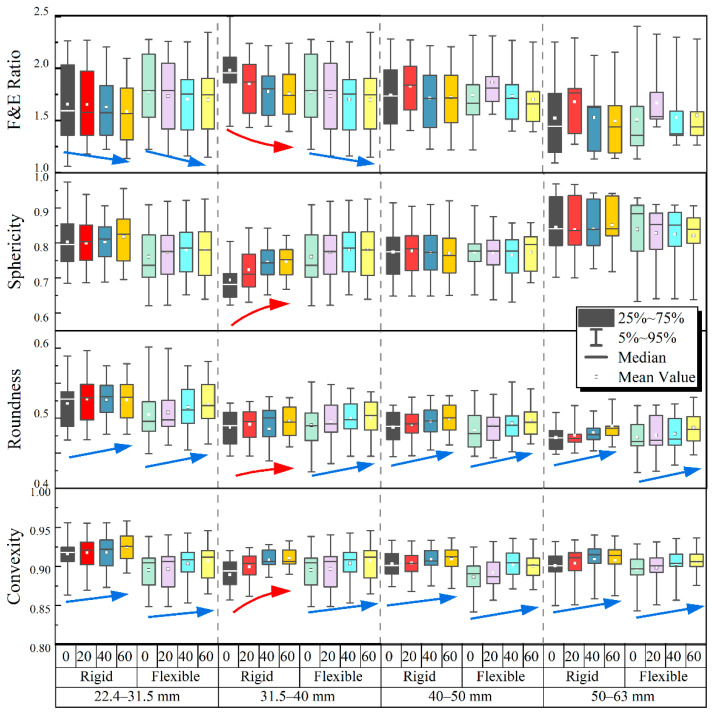
The statistical box plots showing the variations in four shape indices with the number of impact-load applications for particles within different size ranges of multiple-size ballast specimens over rigid and flexible supports.

Figure 16 demonstrates, through statistical box plots, the variations in the four shape indices with the number of impact-load applications for particles within different size ranges of multiple-size ballast specimens over rigid and flexible supports (where the arrows in different colors indicate the trends of shape index variations). As can be seen from Figure 16, the variations in all four shape indices corresponding to the rigid and flexible under-ballast supports exhibit approximately the same pattern. As the number of impact-load applications increases, the F & E ratio gradually decreases and the sphericity gradually increases, indicating that the overall morphology of the ballast particles gradually tends to be spherical. The roundness of the ballast particles gradually increases under impact loading, which indicates that the ballast particles gradually become smooth and the angularity decreases. The convexity increases gradually with an increasing number of impact-load applications (i.e., the concaveness of the ballast particles reduces, and the interparticle contacts become more concentrated). In addition, among the four different particle size ranges, the shape indices of the ballast particles within the size range of 31.5–40 mm exhibit the most profound changes. This may imply that ballast particles within this particular size range are prone to experience the most severe degradation under the applications of impact loading, partly because the relatively irregular shape (high F & E ratio and low sphericity) was initially observed for such particles prior to the impact-load tests. Both the F & E ratio and sphericity indices of the large-size ballast particles (i.e., 40–63 mm) fluctuate with the increasing number of impact-load applications, thus suggesting that they may not be suitable for describing particle-shape degradation, and especially for large particles. By examining Figure 16 more carefully, it can also be found that ballast particles within the size range of 50–63 mm possess a relatively more regular shape (i.e., greater sphericity and lower F & E ratio values) than the others, which may partly explain why they are more difficult to break.

### 3.3. Degradation Trends and Estimation Models of Particle Shape and Breakage

According to the above results and analysis, both the particle shape and size affect ballast degradation (abrasion and crushing) under impact loading, with a more significant influence exerted by the particle shape. Specifically, the breakage of flake-like particles under impact loading is about 1.5–1.66 times higher than that of regular particles, and 1.25–1.5 times higher than that of needle-like particles. This result shares reasonably consistent trends with the Los Angeles abrasion (LAA) test results reported by Guo et al. [21], (i.e., the LAA rate of the flake- or needle-like particles within the size range of 50–60 mm is 3.63 times greater than that of a cubic shape, 3.59 times greater than that of 40–50 mm, and 1.39 times greater than that of 25–35 mm). Note that the inconsistency in the specific numbers and/or orders of magnitude may be attributable to the distinct working mechanisms of the two tests. In addition, the ballast particles over rigid support experienced more severe breakage than those over flexible support. This trend also agrees reasonably well with previous studies [15,16].

**Figure 17:**
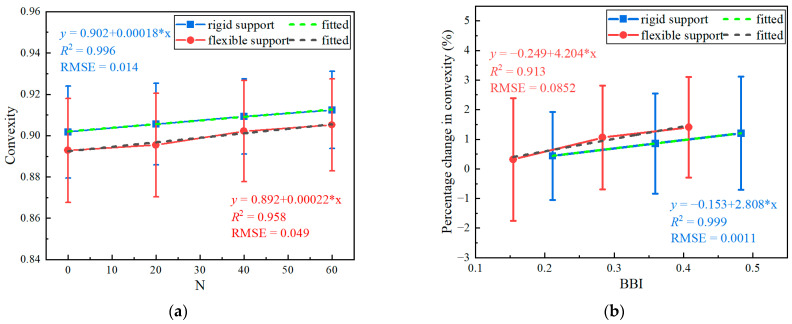
The evolution of (**a**) convexity index with the number of impact-load applications (N), and (**b**) the statistical correlation of the breakage index (BBI) with the convexity index for multiple-size ballasts over rigid and flexible supports.

On the basis of the aforementioned laboratory impact-load-test results, extra efforts were paid to predict the degradation rates of the ballast particle shape and breakage. This could contribute significantly to the transition of routine ballast-bed-maintenance operations (e.g., ballast cleaning and replacing) from the current plan-based scheduling to condition-based upgrading. We attempted to fit the convexity index, previously found in this study to be suitable for characterizing ballast degradation trends, against the number of impact-load applications (N) and the breakage index (BBI). For brevity and illustration purposes, only the average values were analyzed, as shown in Figure 17. Although limited by the small-size datasets obtained in this study, the statically fitted equations in Figure 17 can still serve as the benchmark references for the empirical estimation of the ballast particle shape and breakage degradation trends. Once sufficiently big data from laboratory and field tests are established in the near future, they can be further finetuned and enhanced to guide cost-effective ballast-bed-maintenance management.

## 4. Summary and Conclusions

In this paper, the effects of the particle size and shape, impact energy, and under-ballast support on the particle degradation (abrasion and breakage) evolution characteristics were investigated by conducting laboratory impact-load tests on single- and multiple-size ballasts. The morphology of the ballast particles subjected to impact loading were quantified from 3D-laser-scanning and digital-image-processing techniques. The following major conclusions were obtained:

- Both the particle size and shape affect ballast breakage, with a more profound influence exerted by the particle shape. The larger-size particles exhibit more severe breakage and a greater ultimate breakage limit. The breakage of flake-like particles under impact loading is about 1.5–1.66 times higher than that of regular particles, and 1.25–1.5 times higher than that of needle-like particles;
- Under impact loading, small-size ballast particles are more likely to generate fine particles, while large-size ballast particles degrade mainly with sharp corners fractured. The flake-like particles produce more fine particles during the degradation process (e.g., the modified fouling index (FI) of flake-like ballast particles within 31.5–40 mm is about 3.6 times greater than that of regular particles within 50–63 mm);
- Ballast particles over rigid support are more prone to breakage than those over flexible support, with this difference escalated by the increasing impact energy (e.g., the severity of the ballast breakage and fines generation (quantified by the modified FI) increases by 50% and 74% due to the increase in the under-ballast support stiffness by 100 times, and the drop height of 80 cm, respectively). Therefore, the degradation of ballast beds underlain by rigid support (e.g., bridge decks) should be emphasized, and especially for increased axle loads and train speeds;
- The ballast particles gradually tend to become spherical under impact loading, with the angularity and concavity decreasing. The convexity and ballast breakage index (BBI) are promising for describing particle degradation, and their statistical correlation is potentially useful for the transition of ballast-bed-maintenance management from the current plan-based scheduling to condition-based upgrading.

## Figures and Tables

**Table 1:** The impact-load-test matrix for single-size ballast particles.

Specimen No.	Size Range (mm)	Shape Category	Drop Height of Hammer (cm)	Under-Ballast Support
1	31.5–40	Regular	60	Concrete block
2		Flake-like		
3		Needle-like		
4	40–50	Regular	60	Concrete block
5		Flake-like		
6		Needle-like		
7	50–63	Regular	60	Concrete block
8		Needle-like		

**Table 2:** The impact-load-test matrix for multiple-size ballasts.

Specimen No.	Size Range (mm)	Shape Category **	Under-Ballast Support	Drop Height (cm)
1	22.4–63	Regular	Concrete block	20
2				40
3 *				60
4				80
5	22.4–63	Regular	Sand layer	20
6				40
7 *				60
8				80

Note: The superscript “*” denotes that the ballast particles of the related specimens were scanned for shape quantification, and the superscript “**” denotes that the majority of ballast particles are of a regular shape, as the contents of the needle- and flake-like particles were controlled within 10%.

**Table 3:** The fitted coefficient values of Equations (7) and (9) for distinctly shaped single-size ballasts.

Breakage Index	Size-Range (mm) and Shape Coefficients	Regular Shape			Needle-like			Flake-like	
		31.5–40	40–50	50–63	31.5–40	40–50	50–63	31.5–40	40–50
$B_{g}$	a	30.59168	30.7347	58.96191	64.65747	60.25032	79.06351	67.05557	72.76099
	b	0.01732	0.0237	0.001188	0.01387	0.02406	0.01752	0.03596	0.04695
	$R^{2}$	0.99262	0.95883	0.98892	0.99461	0.97851	0.9847	0.9976	0.99691
BBI	a	0.47296	0.34211	0.46998	0.56019	0.60622	0.72009	0.75096	0.7896
	b	0.01054	0.01567	0.01216	0.01667	0.01803	0.01662	0.02788	0.03019
	$R^{2}$	0.98952	0.99219	0.99748	0.99847	0.99497	0.99906	0.99921	0.99926
Modified FI	a	0.28907	−0.16959	0.00781	0.13897	0.19846	0.19026	0.45867	0.18457
	*k*	0.0652	0.05712	0.03767	0.08102	0.04852	0.05167	0.1151	0.09415
	$R^{2}$	0.97918	0.986	0.99656	0.98976	0.95546	0.97127	0.97721	0.98536

**Table 4:** The fitted coefficient values of Equations (7) and (9) for multiple-size ballasts under different combinations of under-ballast supports and drop heights.

Breakage Index		Fitted Coefficients	Rigid Support (Concrete Block)				Flexible Support (Sand)			
	Drop Height		20 cm	40 cm	60 cm	80 cm	20 cm	40 cm	60 cm	80 cm
$B_{g}$	a		7.93047	28.35468	38.44433	43.91049	8.73478	15.23696	30.02918	36.27785
	b		0.02631	0.01235	0.01246	0.02757	0.03404	0.0207	0.01177	0.01236
	$R^{2}$		0.73699	0.99234	0.97842	0.99944	0.99825	0.95393	0.99555	0.95768
BBI	a		0.50518	0.78741	0.86425	1.24718	0.42986	0.78046	0.82467	0.83136
	b		0.00953	0.01015	0.014	0.02121	0.01073	0.00941	0.01102	0.0156
	$R^{2}$		0.98103	0.99792	0.99881	0.99814	0.98626	0.99848	0.99568	0.99769
D	a		1.63734	1.69482	1.75873	1.9448	1.45094	1.65577	1.71411	1.81566
	b		0.07974	0.10696	0.1531	0.14758	0.14756	0.106	0.12839	0.14887
	$R^{2}$		0.98295	0.96913	0.96305	0.98492	0.98888	0.98789	0.99155	0.98618
Modified FI	a		−0.13856	−0.06773	−0.09337	0.17198	0.12339	0.00823	0.2094	0.20149
	*k*		0.03629	0.05657	0.08129	0.14783	0.01972	0.04291	0.05675	0.08479
	$R^{2}$		0.95401	0.9572	0.93278	0.995	0.94759	0.98817	0.95458	0.98899

## Data Availability

The data will be made available upon reasonable request to the corresponding author.
